# Inhibition of the Growth Factor MDK/Midkine by a Novel Small Molecule Compound to Treat Non-Small Cell Lung Cancer

**DOI:** 10.1371/journal.pone.0071093

**Published:** 2013-08-16

**Authors:** Huifang Hao, Yutaka Maeda, Takuya Fukazawa, Tomoki Yamatsuji, Munenori Takaoka, Xiao-Hong Bao, Junji Matsuoka, Tatsuo Okui, Tsuyoshi Shimo, Nagio Takigawa, Yasuko Tomono, Motowo Nakajima, Iris M. Fink-Baldauf, Sandra Nelson, William Seibel, Ruben Papoian, Jeffrey A. Whitsett, Yoshio Naomoto

**Affiliations:** 1 Kawasaki Hospital Research Center, Kawasaki Medical School, Okayama, Japan; 2 Department of General Surgery, Kawasaki Medical School, Okayama, Japan; 3 Department of General Internal Medicine 4, Kawasaki Medical School, Okayama, Japan; 4 Division of Pulmonary Biology, Cincinnati Children's Hospital Medical Center, Cincinnati, Ohio, United States of America; 5 Department of Palliative Care and Cancer Survivorship, Okayama University Graduate School of Medicine, Dentistry and Pharmaceutical Sciences, Okayama, Japan; 6 Department of Oral and Maxillofacial Surgery, Okayama University Graduate School of Medicine, Dentistry and Pharmaceutical Sciences, Okayama, Japan; 7 Shigei Medical Research Institute, Okayama, Japan; 8 SBI Pharmaceuticals Co., Ltd., Tokyo, Japan; 9 Drug Discovery Center, University of Cincinnati, Cincinnati Ohio, United States of America; 10 Department of Biochemistry, School of Basical Medicine, Liaoning Medical University, Jinzhou, China; National Cancer Center, Japan

## Abstract

Midkine (MDK) is a heparin-binding growth factor that is highly expressed in many malignant tumors, including lung cancers. MDK activates the PI3K pathway and induces anti-apoptotic activity, in turn enhancing the survival of tumors. Therefore, the inhibition of MDK is considered a potential strategy for cancer therapy. In the present study, we demonstrate a novel small molecule compound (iMDK) that targets MDK. iMDK inhibited the cell growth of MDK-positive H441 lung adenocarcinoma cells that harbor an oncogenic *KRAS* mutation and H520 squamous cell lung cancer cells, both of which are types of untreatable lung cancer. However, iMDK did not reduce the cell viability of MDK-negative A549 lung adenocarcinoma cells or normal human lung fibroblast (NHLF) cells indicating its specificity. iMDK suppressed the endogenous expression of MDK but not that of other growth factors such as PTN or VEGF. iMDK suppressed the growth of H441 cells by inhibiting the PI3K pathway and inducing apoptosis. Systemic administration of iMDK significantly inhibited tumor growth in a xenograft mouse model *in vivo*. Inhibition of MDK with iMDK provides a potential therapeutic approach for the treatment of lung cancers that are driven by MDK.

## Introduction

Lung cancer is the leading cause of cancer-related mortality worldwide [1,2]. Conventional chemotherapeutic regimens target lung cancer cells but also normal proliferating cells. Presently, survival following conventional chemotherapy of lung adenocarcinoma (the most frequent type of lung cancer) provides less than one-year median survival from the time of diagnosis [3]. Molecular pathway-specific therapies for lung adenocarcinoma, e.g., targeting mutant *EGFR* or *ALK* fusions, limit non-tumor toxicity and extend survival time compared to the conventional chemotherapies [4]–[6]. However, there is no molecularly targeted therapy for mutant *KRAS*-driven lung adenocarcinoma, the most frequent type of lung adenocarcinoma in the Caucasian population. Moreover, effective molecularly targeted therapies have been developed for adenocarcinomas but not squamous cell carcinomas. Therefore, specific therapies that target various lung tumor types are desperately needed [7]–[9].

Midkine (MDK) is a heparin-binding growth factor that is highly expressed in many malignant tumors, including lung, esophageal, stomach, colon, hepatocellular, breast, renal and pancreatic carcinoma [10]–[15]. MDK binds to multiple membrane receptors, ALK, syndecans, PTP and LRP, and subsequently activates the PI3 kinase (PI3K) and MAP kinase pathways. These two pathways induce cell proliferation and enhance angiogenic and anti-apoptotic activities [16], [17]. Inhibition of *MDK* by siRNA suppresses cell growth of cancer cells that express MDK [18], indicating that MDK might be a potential target for lung cancer therapy. Since mice lacking the *Mdk* gene are viable [19], targeting MDK is an attractive therapeutic approach since its inhibition is unlikely to have systemic deleterious effects. The recognition of the potential role of the MDK pathway in the treatment of cancer has increased efforts to identify MDK inhibitors. Matsui et al. identified synthetic peptides and compounds that inhibit MDK-mediated cell migration *in vitro*; however, these proved not to be potent and lack clinical utility [20].

In the present study, we identified a low molecular weight compound (iMDK) that suppressed endogenous MDK expression. iMDK inhibited the growth of MDK-expressing H441 lung adenocarcinoma cells that harbor an oncogenic *KRAS* mutation and H520 squamous cell lung cancer cells *in vitro*. Moreover, iMDK suppressed lung tumor growth and induced cell death by inhibiting the PI3 kinase pathway and inducing apoptosis in a xenograft mouse model derived from H441 lung adenocarcinoma cells. Targeting the expression of MDK provides a new therapeutic approach for the treatment of MDK-expressing non-small cell lung cancers.

## Materials and Methods

### Reagents

3-[2-(4-fluorobenzyl)imidazo [2,1-beta][1], [3]thiazol-6-yl]-2H-chromen-2-one (hereafter iMDK) purchased from ChemDiv (San Diego, CA) was dissolved in DMSO. PD0325901 (a MEK inhibitor) was obtained from signalinginhibitors.com (Wedel, Schleswig Holstein, Germany).

### Cell Lines and Culture Conditions

The human pulmonary adenocarcinoma cells H322, H358, H441 and A549 and the human lung squamous cell carcinoma cells H520 were obtained from the American Type Culture Collection (Manassas, VA) and grown in RPMI 1640 (H322, H358, H520) or high glucose Dulbecco's modified Eagle medium (H441, A549 cells) supplemented with 10% heat-inactivated fetal bovine serum. The human malignant mesothelioma cells ACC-MESO-1 (MESO-1) obtained from JCRB Cell Bank (Osaka, Japan) and the human lung squamous cell carcinoma cells SQ5 kindly provided from by Dr. Kiura Katsuyuki (Department of Hematology, Oncology, and Respiratory Medicine, Okayama University Graduate School of Medicine, Dentistry, and Pharmaceutical Sciences, Okayama, Japan) [21] were grown in RPMI 1640 supplemented with 10% heat-inactivated fetal bovine serum. The normal human lung fibroblasts NHLF cells obtained from Clonetics (San Diego, CA) were grown in high glucose Dulbecco's modified Eagle medium. All cell lines were cultured in 5% CO_2_ at 37°C.

### Immunoblot analysis

Cells were lysed in ice-cold M-PER lysis buffer purchased from Thermo Fisher Scientific (Rockford, IL). Cell lysates were clarified by centrifugation (20 min at 15,000×g at 4°C) and protein concentration determined using the BCA protein assay (Thermo Scientific, Rockford, IL). Equal amounts of protein were separated on an SDS-PAGE gel. The gel was electrophoretically transferred to a Hybond PVDF transfer membrane (GE Healthcare Ltd., Piscataway, NJ) and incubated with primary and secondary antibodies according to the Supersignal^®^ West Pico chemiluminescence protocol (Pierce, Rockford, IL). Antibody specific for β-actin antibody was obtained from Sigma (St. Louis, MO) and antibody specific for human MDK and PTN (Pleiotrophin) were obtained from Abcam (Cambridge, UK). Antibody specific for caspase-3, PI3 kinase p85, phosphorylated-PI3K p85 (Tyr458)/p55 (Tyr199), AKT, phosphorylated-AKT (Ser473), ERK1/2, phosphorylated-ERK1/2 (Thr202/Tyr204), p38MAPK, phosphorylated-p38 MAPK (Thr180/yr182), Bad, XIAP, survivin were obtained from Cell Signaling Technology (Beverly, MA). Antibody specific for VEGF was purchased from Santa Cruz Biotechnology (Santa Cruz, CA). Secondary horseradish peroxidase-conjugated antibodies were obtained from Jackson Immunoresearch Laboratories (West Grove, PA).

### siRNA mediated inhibition of MDK

**A.** H441 cells were plated in a 12-well plate at a density of 1×10^5^ per well and cultured overnight at 37°C. The following day 100 pmol of two different siRNAs targeting MDK (D-003677-02-0050 and D-003677-03-0050) or nontargeting siRNA (Thermo Scientific) was transfected using 2 μl Lipofectamine 2000 (Invitrogen Life Technologies, Carlsbad, CA) according to the manufacturer's instructions. Incubation time for transfection reagents was 24 hours, at which time medium was replaced with fresh regular medium. Cells were harvested 48 hours after transfection for immunoblotting and cell growth assays.

### Cell viability assay

H441 cells, A549 cells, H520 cells, HEK293 cells and NHLF cells were plated in 24-well plates at a density of 1×10^5^ cells and cultured at 37°C for 24 hours. Medium was removed by aspiration and replaced with fresh culture medium containing iMDK dissolved in DMSO (10, 50, 100, 500 nmol/L). DMSO alone was used as the control. Cells were treated for 48 hours and collected by trypsinization. Viable cells were assessed by trypan blue exclusion and WST-1 assays (Roche Molecular Biochemicals, Laval, Quebec, Canada) according to the manufacturer's protocol. Recombinant MDK was purchased from R&D Systems (Minneapolis, MN) and reconstituted in water for block experiments. For MDK block experiments, H441 cells were cultured in Dulbecco's modified Eagle medium supplemented with 1% heat-inactivated fetal bovine overnight and recombinant MDK (25 nM) and/or iMDK (25 nM) was added for an additional 48 hours.

### Hoechst staining

Morphologic characteristics of apoptosis were evaluated using Hoechst 33342 dye (Molecular Probes, Eugene, OR), which stains DNA. Cells were incubated with 1 **μ**g/ml Hoechst dye and then visualized under a fluorescence microscope (IX81, Olympus Medical Systems Corp., Tokyo, Japan) [22].

### TUNEL staining

Terminal deoxynucleotidyltransferase-mediated dUTP-biotin nick end labeling (TUNEL) staining was performed to detect apoptosis using the DeadEnd colormetric TUNEL system (Promega, Madison, WI) according to the manufacturer's protocol.

### Flow cytometric analysis for apoptosis

Cells were plated in 24-well plates at a density of 0.5×10^5^ cells per well 1 day before the treatments. After 72 hours, cells were harvested and washed once with PBS. Cells were resuspended in PBS containing 0.2% Triton X-100 and 1 mg/ml RNase for 5 min at room temperature and then stained with propidium iodide at 50 μg/ml to determine sub-G_0_/G_1_ DNA content using a FACScan. Doublets, cell debris, and fixation artifacts were gated out, and sub-G_0_/G_1_ DNA content was determined using Cell Quest Ver. 3.3 software.

### Mouse experiments

The experimental protocol was approved by the Ethics Review Committee for Animal Experimentation of Okayama University Graduate School of Medicine and Dentistry (Ethics Committee reference number: OKU-2011472). Human lung cancer xenografts were established in 6-wk-old female BALB/c nude mice (Charles River Laboratories Japan, Kanagawa, Japan) by subcutaneous (s.c.) inoculation of H441 cells (1×10^6^/50 μl) mixed with Matrigel^®^ (BD Pharmingen, San Diego, CA; 50 μl) into the dorsal flank [23]. The mice were randomly assigned into three groups (n = 8 per group) 14 days after tumor inoculations. One group of mice was intraperitonially treated with 100 μl solution containing iMDK (9 mg/kg) three days per week (on days 1, 3, 5, 8, 10) and another group of mice was treated five days per week (on days 1, 2, 3, 4, 5, 8, 9, 10). DMSO was administered into the control group. Tumors were measured two to three times a week, and tumor volume was calculated as a × b^2^×0.5, where a and b were large and small diameters, respectively. On day 10, body weight was measured and all mice were then sacrificed and tumors removed and prepared for histology. For analysis of serum AST and ALT, blood was drawn from the heart 48 hours after the treatment with iMDK (9 mg/kg).

For immunohistochemistry, sections were sequentially deparaffinized through a series of xylene, graded ethanol, and water immersion steps. After being autoclaved in 0.2% citrate buffer for 15 minutes, sections were incubated with 3% hydrogen peroxide for 30 minutes to block endogenous peroxidase activity. A primary antibody specific for phosphorylated-PI3K p85 (Tyr458)/p55 (Tyr199) was obtained from Cell Signaling Technology. The antibodies for MDK and CD31/PECAM-1 were from Abcam. The antibody for VEGF was from Santa Cruz Biotechnology (Santa Cruz, CA). Specimens were incubated overnight at 4°C with a 1∶200 dilution of antibody followed by three washes with TBS. The slides were treated with streptavidin-biotin complex (Envision System labeled polymer, horseradish peroxidase [HRP], Dako, Carpinteria, CA) for 60 minutes at a dilution of 1∶100. Immunoreactions were visualized using a 3,3′-diaminobenzidine (DAB) substrate-chromogen solution (Dako Cytomation Liquid DAB Substrate Chromogen System, Dako) and counterstained with hematoxylin. Sections were immersed in an ethanol and xylene bath and mounted for examination.

### Statistical analysis

Statistically significant differences between means and medians of the study groups were evaluated using Student's t-test. Statistical significance was defined as p<0.05 (#) or p<0.01 (*).

## Results

### Inhibition of MDK reduces viability of H441 lung adenocarcinoma cells

**Figure 1:**
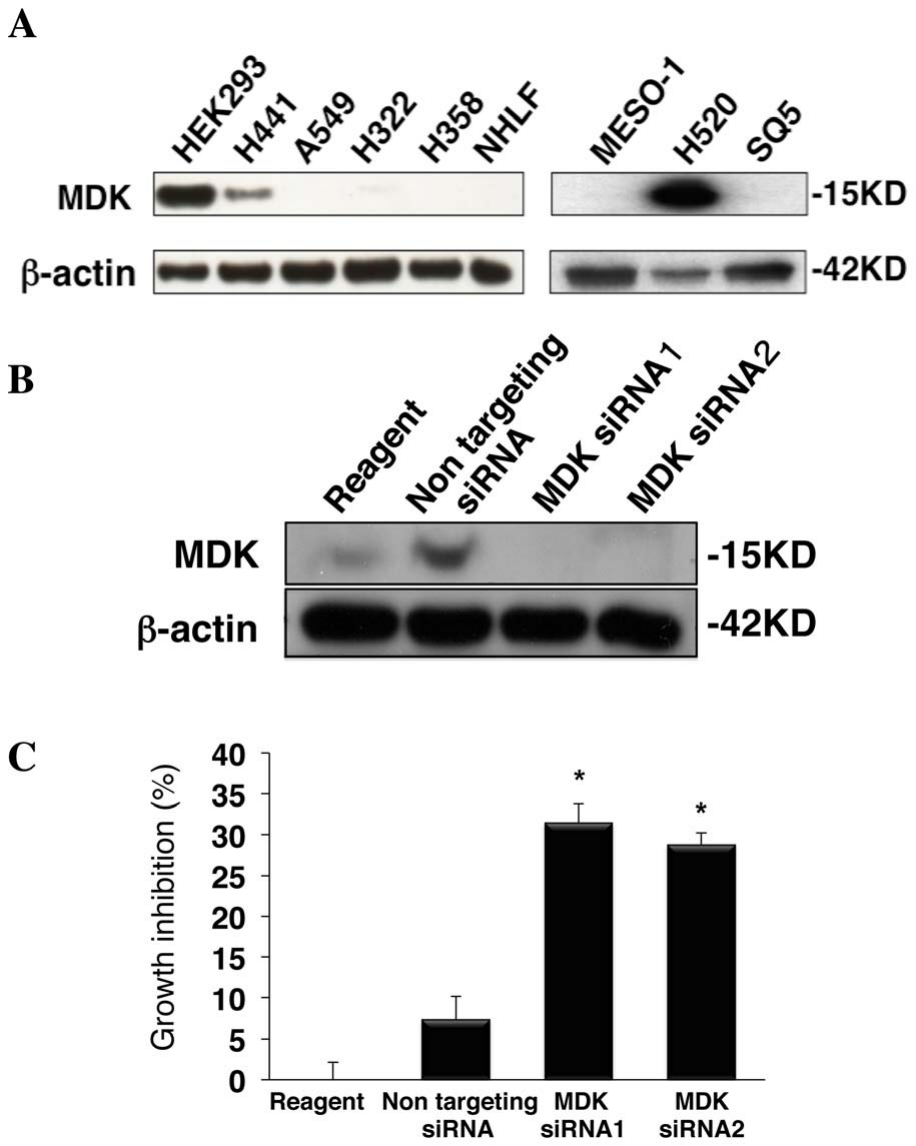
Growth inhibition was increased by the MDK knockdown in H441 lung adenocarcinoma cells. MDK was detected in HEK293 cells, H441 lung adenocarcinoma cells and H520 human lung squamous cell carcinoma cells but not in the other kinds of cells including NHLF (Normal Human Lung Fibroblast) cells. Protein expression of MDK and **μ**-actin was confirmed by immunoblot as described in Methods. **A.** MDK was suppressed by two different MDK siRNAs (MDK siRNA1 and MDK siRNA2) in H441 cells. Protein expression was confirmed by immunoblot as described in A. **B.** Growth inhibition in H441 cells after MDK gene silencing was significantly increased. Cell viability was assessed by trypan blue exclusion assay as described in Methods. Statistical significance was defined as p<0.01 (*).

In order to find a NSCLC cell line that is dependent on MDK for cell growth, we assessed the endogenous expression of MDK protein in four different NSCLC cell l ines and NHLF (Normal Human Lung Fibroblast) cells. HEK293 embryonic kidney cells were used as a positive control for MDK expression. As shown in Figure 1A, MDK was detected in HEK293 cells, H441 lung adenocarcinoma cells and H520 human lung squamous cell carcinoma cells but not in A549, H322 and H358 lung adenocarcinoma cells, SQ5 lung squamous cell carcinoma cells or MESO-1 malignant mesothelioma cells. MDK was not expressed in normal NHLF cells. The H441 cell line is derived from a NSCLC lung tumor that has a *KRAS* mutation. Activated *RAS* is the most common mutation associated with pulmonary adenocarcinomas in the Caucasian population and effective treatments for this disease have not yet been identified [24]. In order to determine whether H441 cells depend on MDK for cell viability, we inhibited MDK using siRNA and examined cell growth in the presence and absence of MDK. As shown in Figure 1B, 48 hours after transfection, two different MDK siRNAs suppressed MDK in H441 cells compared to non-targeting siRNA. Growth inhibition was significantly induced by the suppression of MDK (p<0.01; Figure 1C). These results demonstrate that targeting MDK is an effective strategy for suppressing cell growth of MDK-expressing non-small cell lung cancer.

### iMDK inhibits endogenous MDK expression in H441 lung adenocarcinoma cells

**Figure 2:**
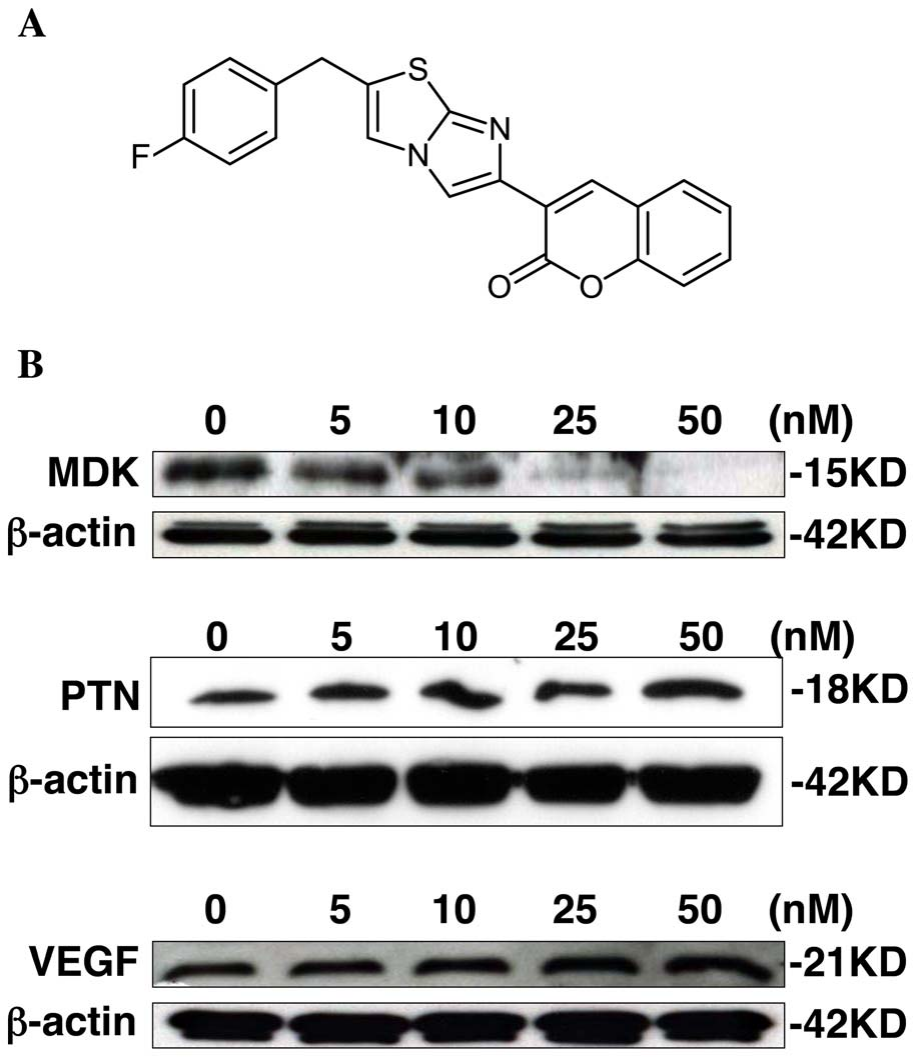
iMDK inhibited the expression of MDK in H441 pulmonary adenocarcinoma cells. **A.** Structure of iMDK (3-[2-(4-fluorobenzyl)imidazo [2,1-beta][1], [3]thiazol-6-yl]-2H-chromen-2-one). **B.** MDK but not PTN or VEGF was suppressed by iMDK dose-dependently in H441 cells 48 hours after treatment. Immunoblot analysis was performed as described in Methods.

In order to find a therapeutic compound that inhibits MDK expression, we first developed a MDK reporter cell line by stably transfecting HEK293 cells with a MDK promoter-fused luciferase construct. We used this modified cell line to screen 44,000 compounds at the Drug Discovery Center at the University of Cincinnati. Detection of luciferase activity was used to identify MDK inhibitors. In this screening, we identified a compound (3-[2-(4-fluorobenzyl)imidazo[2,1-beta][1], [3]thiazol-6-yl]-2H-chromen-2-one; hereafter iMDK, Figure 2A) that reproducibly inhibited endogenous MDK protein expre ssion. We assessed the effectiveness of iMDK for its ability to specifically inhibit the expression of MDK in H441 cells. As shown in Figure 2B, iMDK inhibited endogenous MDK in a dose-dependent fashion but did not inhibit PTN (Pleiotrophin), which has considerable homology to MDK [17]. Nor did iMDK inhibit another growth factor, VEGF, in H441 lung adenocarcinoma cells 48 hours after treatment.

### iMDK inhibits cell viability of MDK-expressing non-small cell lung carcinoma cells

**Figure 3:**
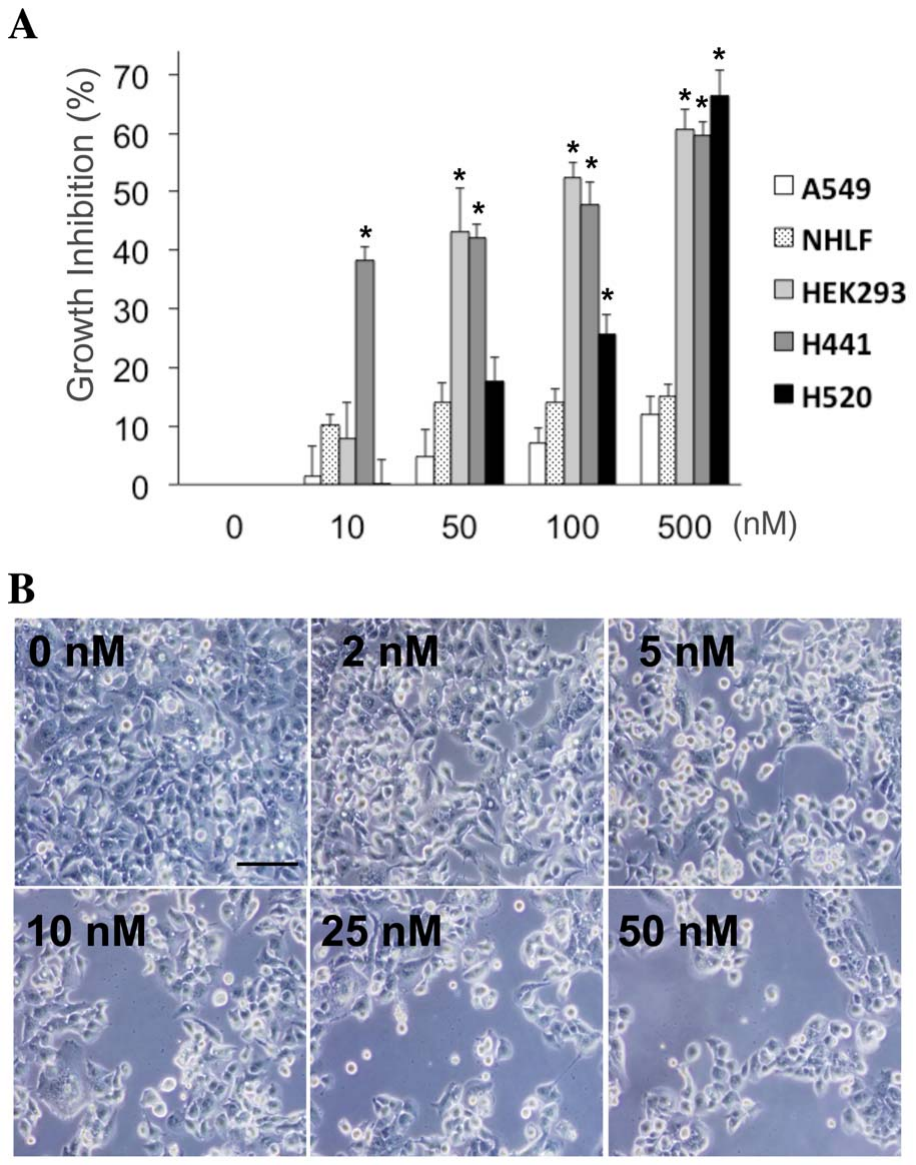
iMDK induced growth inhibition in MDK-positive non-small cell lung carcinoma cells. **B.** Growth inhibition by iMDK was increased in the MDK-positive HEK293, H441 and H520 cells but not the MDK-negative A549 cells or normal NHLF cells after 48 hours of treatment. Cell viability was assessed by trypan blue exclusion assay as described in Methods. Statistical significance was defined as p<0.01 (*). Dose-dependent growth inhibition by iMDK was observed morphologically in H441 lung adenocarcinoma cells. Shown are phase-contrast photomicrographs of H441 cells 48 hours after iMDK treatment (scale bar shows 100 μm).

To further determine the effectiveness and specificity of iMDK in suppressing MDK-expressing tumor cells, we treated both MDK-positive and MDK-negative cells with iMDK and assessed cell viability. MDK is expressed in HEK293 embryonic kidney cells, H441 lung adenocarcinoma cells and H520 lung squamous cell carcinoma cells but not in A549 lung carcinoma cells or non-transformed NHLF cells (Figure 1A). These five cell lines were treated with a range of iMDK concentrations (0 to 500 nM) and cell viability was assessed 48 hours after treatment. Growth inhibition by iMDK was dose-dependently induced in the MDK-positive HEK293, H441 and H520 cells but not the MDK-negative A549 cells or non-transformed NHLF cells (Figure 3A, S1). Morphologically, growth suppression of H441 cells was dose-dependently observed 48 hours after treatment with iMDK (Figure 3B). The suppression of cell growth by iMDK was partially blocked by pretreatment with recombinant MDK (25 nM) in the MDK-positive H441 cells; however, pretreatment with recombinant MDK did not significantly alter cell growth in the MDK-negative A549 cells (Figure S2). This finding suggests that the suppressive effect on cell growth by iMDK is mediated at least in part by the inhibition of MDK expression.

### iMDK induces apoptosis in H441 lung adenocarcinoma cells

**Figure 4:**
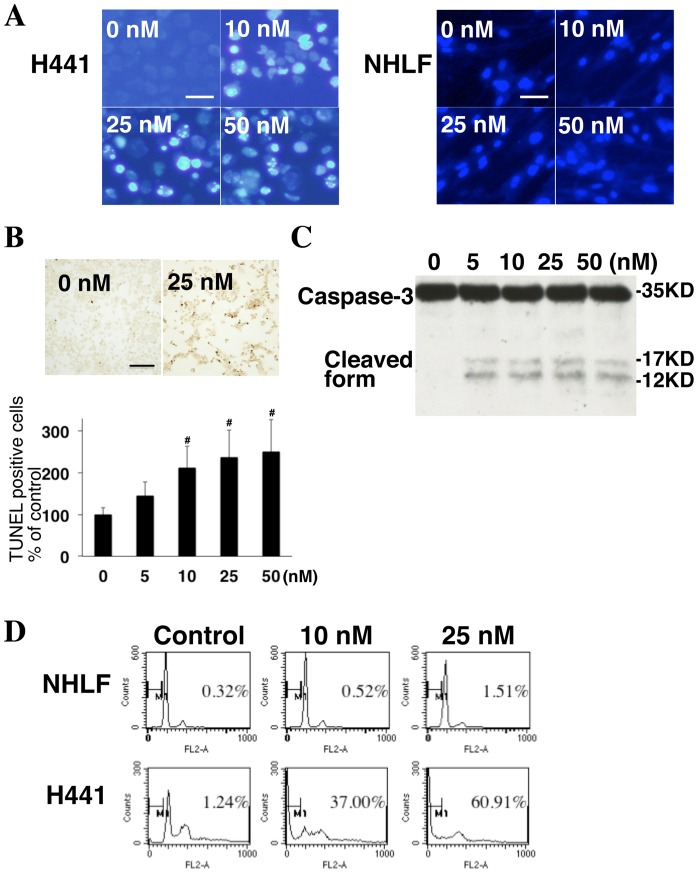
iMDK induced apoptosis in H441 lung adenocarcinoma cells. iMDK dose-dependently increased apoptosis in H441 cells but not normal NHLF cells. Cells were treated with the indicated concentrations of iMDK for 72 hours and then stained with Hoechst 33342 dye and analyzed under a fluorescence microscope as described in Methods (scale bar shows 50 μm). Apoptosis was induced in H441 cells 48 hours after iMDK treatment at a concentration of 25 nM (upper panel, scale bar shows 200 μm). TUNEL positive cells were increased by iMDK treatment in a dose–dependent manner (bottom panel). TUNEL staining was performed as described in Methods. Statistical significance was defined as p<0.05 (#). **A.** Cleaved caspase-3, an apoptosis marker, was increased in H441 cells following iMDK treatment. H441 cells were treated for 48 hours with iMDK in the indicated concentrations and harvesting for immunoblot analysis as described in Methods. **D.** iMDK induced sub-G_0_/G_1_ DNA content in H441 cells. Cells were treated with iMDK for 72 hours and DNA content was measured by propidium iodide stain and flow cytometric analysis as described in Methods.

In order to understand the mechanism by which iMDK inhibits the growth of H441 cells, we assessed the cells for apoptosis 48 hours after treatment with iMDK. As shown in Figure 4A, highly condensed and partly fragmented nuclei were observed in H441 cells but not in normal NHLF cells after the treatment with iMDK, indicating that iMDK induced apoptosis in MDK-expressing H441 cells. TUNEL positive cells were also significantly increased in H441 cells hours after iMDK treatment in a dose-dependent manner (Figure 4B), further confirming the induction of apoptosis by iMDK. As shown in Figure 4C, cleaved forms of caspase-3, a marker of apoptosis was induced by iMDK even at the lowest concentrations (5 nM) in H441 cells 48 hours after treatment. sub-G_0_/G_1_ DNA content of H441 cells was highly increased from 1.24% (DMSO control) to 37.00% (10 nM) and 60.91% (25 nM) 72 hours after treatment with iMDK. However, sub-G_0_G_1_ DNA content of NHLF cells was minimally increased from 0.32% (DMSO control) to 0.52% (10 nM) and 1.51% (25 nM) after treatment with iMDK (Figure 4D). Collectively, these results indicate that iMDK selectively induces apoptosis in MDK-expressing H441 lung adenocarcinoma cells but not in normal NHLF cells that do not express MDK.

### iMDK suppresses the PI3K and induces the apoptotic pathways in H441 lung adenocarcinoma cells

**Figure 5:**
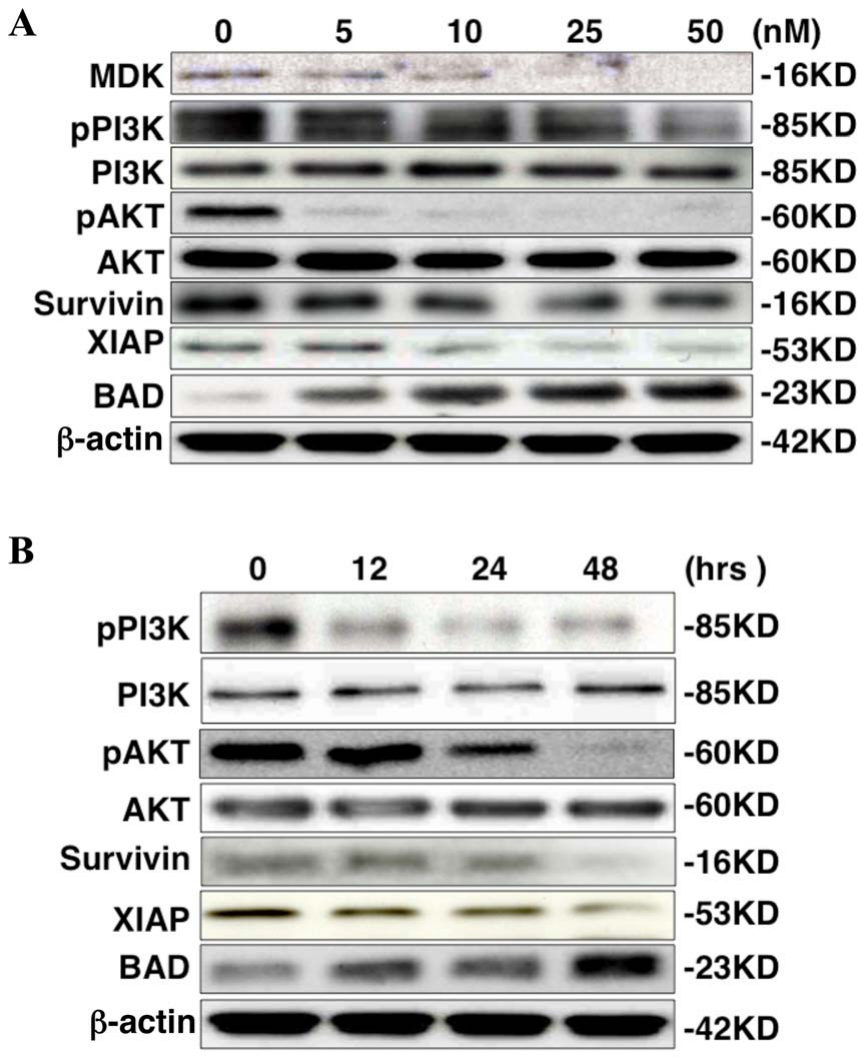
iMDK inhibited the PI3K/AKT pathway and influenced the apoptosis pathway. **A.** Dose-dependently, phosphorylation of PI3K and AKT and the expression of survivin and XIAP, anti-apoptotic factors, were decreased while the expression of BAD, a pro-apoptotic factor, was increased 48 hours after treatment with iMDK. Shown is immunoblot performed as described in Methods. **B.** Time-dependently, phosphorylation of PI3K and AKT and the expression of survivin and XIAP were decreased while the expression of BAD was increased by treatment with iMDK at a concentration of 50 nM. Immunoblot was performed as described in Methods.

PI3K is involved in tumorigenesis by activating AKT, which in turn increases anti-apoptotic factors, such as XIAP and survivin, and decreases a pro-apoptotic factor BAD [25]–[27]. Since MDK activates PI3K activity [16], [28], we sought to determine whether the PI3K pathway was suppressed by iMDK-mediated MDK inhibition in H441 cells. Phosphorylation of PI3K and AKT were suppressed by iMDK in a dose- (Figure 5A) and time-dependent manner (Figure 5B), indicating that the PI3K/AKT pathway is inhibited by iMDK. Anti-apoptotic factors, XIAP and survivin, were reduced while the pro-apoptotic factor, BAD, was induced by iMDK in a dose- and time-dependent fashion (Figure 5), indicating that the apoptotic pathway is induced by iMDK. MDK is also known to activate ERK (a MAPK) in normal non-tumorigenic cells [16], [28]; however, ERK and p38MAPK were not inhibited by iMDK in H441 lung adenocarcinoma cells (Figure S3). These results indicate that iMDK suppresses the PI3K/AKT pathway but not the MAPK pathway and in turn causes apoptosis in H441 cells.

### iMDK suppresses lung tumor growth and induces apoptosis in a xenograft mouse model

**Figure 6:**
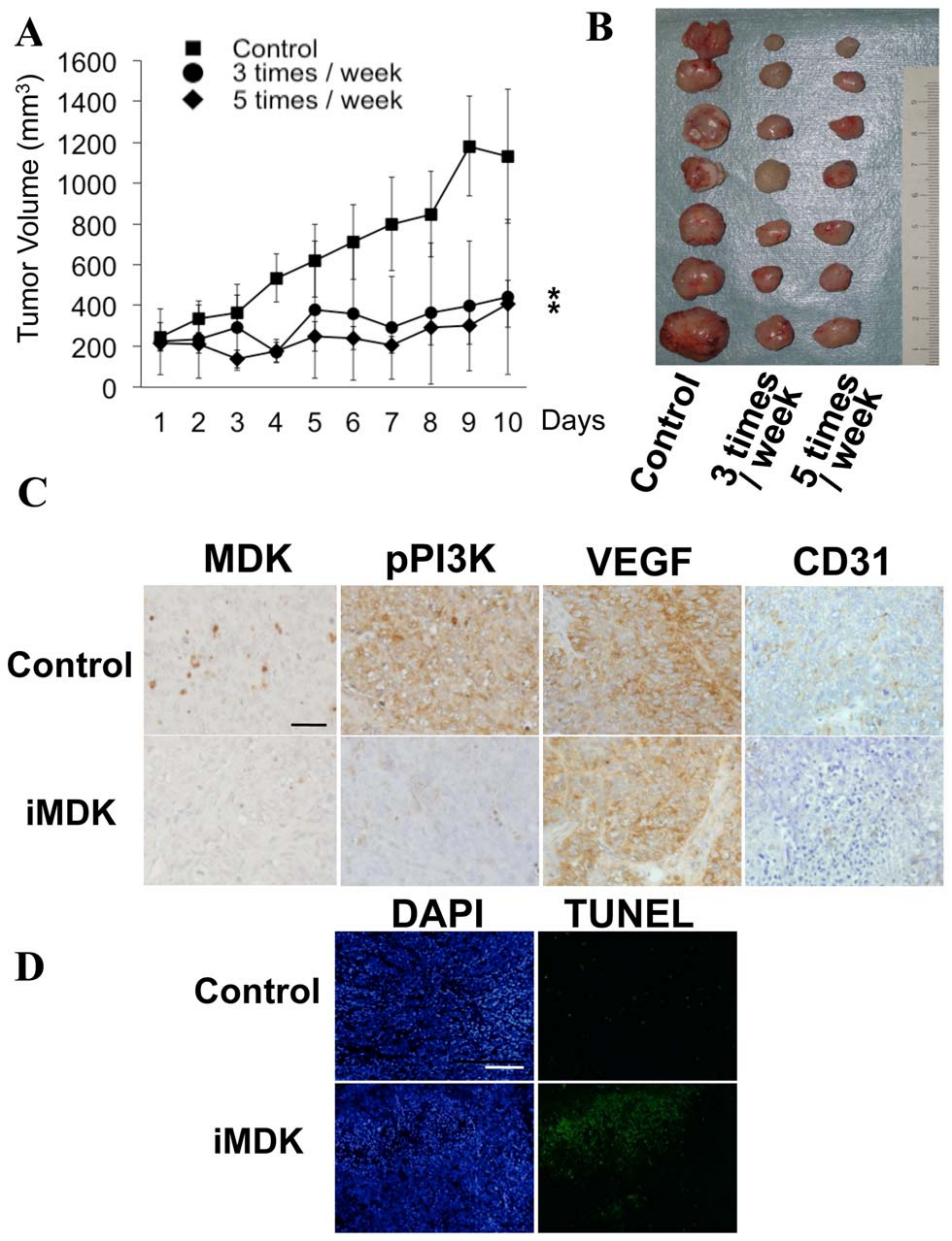
iMDK reduced lung tumor growth in a xenograft mouse model. **A.** Volume of the tumors derived from H441 lung adenocarcinoma cells was reduced after treatment with iMDK (9 mg/kg) in a xenograft mouse model. DMSO was used for the control group. iMDK was administered 3 times/week or 5 times/week as shown. Eight mice were used in each group. Tumor volume was monitored everyday after inoculation of H441 cells. Tumor growth is expressed as mean tumor volume; bars represent SD. Statistical significance was defined as p<0.01 (*). **B.** Shown is an image of xenograft tumors derived from H441 cells, which are dissected from the xenograft mice 10 days after treatment with iMDK. Expression of MDK and phosphorylation of PI3K (pPI3K) were reduced by the iMDK treatment in xenograft tumors. Expression of an angiogenesis marker CD31/PECAM-1 was inhibited by iMDK though expression of an angiogenesis growth factor VEGF was not altered. Immunohistochemistry was performed using the xenograft tumor sections as described in Methods. Control is from the DMSO administered group. (scale bar shows 100 μm). iMDK induced apoptosis *in vivo*. TUNEL staining with the xenograft tumors treated DMSO (control) or iMDK was performed as described in Methods (scale bar shows 200 μm).

In order to determine whether systemic administration of iMDK suppresses tumor growth *in vivo*, iMDK (9 mg/kg) was intraperitoneally injected either 3 or 5 times a week into nude mice bearing xenografts derived from H441 lung adenocarcinoma cells 14 days after tumor inoculation. Lung tumor xenografts continued to grow in the control (DMSO treated) group while lung tumor growth was arrested in iMDK-treated groups. The volume of the tumors in the iMDK-treated group was significantly lower than that in the control group (Figure 6A and 6B). MDK and phosphorylated PI3K were observed in lung tumors of the control mice but not in the iMDK-treated mice (Figure 6C), consistent with *in vitro* studies (Figure 5). DNA fragmentation detected by TUNEL staining was induced in tumors of iMDK-treated mice but not in those from control mice (Figure 6D), indicating that iMDK induces apoptosis *in vivo* as well. Notably, treatment of iMDK did not influence body weight and serum levels of AST and ALT in the mice (Figure S4), suggesting that iMDK does not cause systemic toxicity.

Since MDK is known to induce angiogenesis [29], we sought whether inhibition of angiogenesis by iMDK might in part contribute to the reduction of lung tumors *in vivo*. As shown in Figure 6C, the expression of VEGF, another angiogenesis growth factor [30], was not altered, consistent with *in vitro* study (Figure 2); however, the expression of CD31/PECAM-1, an angiogenesis marker [30], was reduced in lung tumors treated by iMDK, indicating that iMDK inhibits angiogenesis independently of VEGF and in turn suppresses lung tumorigenesis *in vivo*. These *in vivo* results indicate that iMDK is likely to be a promising therapeutic anti-tumorigenic/angiogenic drug targeting MDK-expressing lung cancer without side effects.

## Discussion

The success of small molecules specifically inhibiting EGFR function in mutant *EGFR*-driven lung adenocarcinoma has promoted molecular targeted therapy for lung cancer [4], [5], [31], [32]. On the other hand, targeted treatments for mutant *KRAS*-driven lung adenocarcinoma and lung squamous cell carcinoma have not been developed other than chemotherapy that target both cancer cells and normal proliferating cells [7]–[9]. In the present study, we have identified a small molecule compound iMDK that inhibits the expression of MDK, a tumor-promoting growth factor. iMDK inhibited the PI3K/AKT pathway and suppressed *KRAS*-mutated lung adenocarcinoma by inducing apoptosis *in vitro* and *in vivo*. iMDK did not impair the viability of normal proliferating NHLF cells. Further, no obvious systemic toxicity was observed in iMDK-treated mice, supporting the potential utility of iMDK for therapy of MDK-dependent lung adenocarcinoma.

MDK is known to activate not only the PI3K/AKT pathway but also the MAPK pathway in primary neuronal culture [16] and myocardium [28]; however, in our study iMDK inhibited only the PI3K pathway but not the MAPK pathway, and actually activated the MAPK pathway in H441 lung adenocarcinoma cells (Figure S3). The mechanism by which iMDK inhibits only the PI3K pathway but not the MAPK pathway is unknown. The upregulation of the MAPK pathway might be compensatory activation by the downregulation of the PI3K pathway in the H441 cells. The treatment of H441 cells with the MAPK inhibitor (PD0325901) or the PI3K/AKT inhibitor (LY294002) did not influence the expression of MDK (Figure S5 and data not shown), suggesting that suppression of MDK by iMDK is not mediated through the MAPK and PI3K/AKT pathways.

Since MDK is highly expressed in hepatocellular, gastric, colorectal and prostate cancers [17], the MDK inhibitor iMDK may be useful for treating non-pulmonary tumors as well. Also, MDK expression is associated with various inflammatory diseases, including rheumatoid arthritis and atherosclerosis [19], [33]. *Mdk*-knockout mice are resistant to the development of rheumatoid arthritis by preventing inflammatory leukocyte migration and osteoclast differentiation [33]. The knockout mice are also resistant to neointimal formation, a common feature of atherosclerosis and restenosis [34]. These results suggest that iMDK may be useful for treating these non-tumorigenic diseases as well.

In addition to iMDK, we identified another small molecule compound that also suppressed the expression of MDK. The other compound has a Cl instead of F in the structure of the iMDK molecule and exerts similar dose effect to iMDK (data not shown), indicating that iMDK may be structurally modified to be more effective and safe. Since the extent of inhibition of *MDK* RNA was less than that of MDK protein (data not shown), iMDK may target the MDK protein directly. Biotin-tagged iMDK or radioisotope-labeled iMDK will be required to identify molecules or factors that directly associate with iMDK, which will lead to the understanding of the mechanism(s) by which iMDK inhibits the expression of MDK.

In summary, we have determined that the MDK inhibitor iMDK suppresses non-small cell lung cancer expressing MDK *in vitro* and *in vivo* without harming normal cells. Although further studies are needed, including identification of iMDK direct targets, additional structural modification and safety validation, iMDK looks to be a promising treatment for *KRAS* mutant pulmonary adenocarcinoma and squamous cell carcinoma and possibly for the treatment of other cancers and chronic inflammatory diseases.

## Supporting Information

Figure S1 **Growth inhibition by iMDK was increased in MDK-expressing cells.** iMDK induced growth inhibition in MDK-positive H441 lung carcinoma cells and HEK293 embryonic kidney cells but not in MDK-negative A549 lung carcinoma cells. Cell viability was assessed by the WST-1 assay 48 hours after the iMDK treatment and represented as % growth inhibition as described in Methods. Statistical significance was defined as p<0.05 (#).
(TIF)

Figure S2 **Recombinant MDK rescued iMDK-induced cell viability inhibition in H441 lung adenocarcinoma cells.** **A.** Recombinant MDK (25 nM) blocked the iMDK (25 nM)-mediated cell growth inhibition in MDK-positive H441 lung adenocarcinoma cells. Cell viability was assessed by trypan blue exclusion assay as described in Methods. **B.** Recombinant MDK (25 nM) and/or iMDK (25 nM) did not alter cell growth in MDK-negative A549 lung carcinoma cells. Cell viability was assessed by trypan blue exclusion assay as described in Methods.
(TIF)

Figure S3 **iMDK activated the MAPK pathway in H441 pulmonary adenocarcinoma cells.** Phosphorylation of ERK (a MAPK) and p38MAPK was increased 48 hours after treatment with iMDK at the indicated concentrations in H441 cells. Shown is immunoblot performed as described in Methods.
(TIF)

Figure S4 **Systemic toxicity was not observed after iMDK treatment in BALB/c nude mice.** A. Body weight of the nude mice was not altered by treatment with iMDK (9 mg/kg). The body weight of each mouse group (DMSO administered Control, 3 times/week or 5 times/week) was measured on day 10. Shown is the mean of the body weight (g) from four mice of each group; bars, SD. B. Liver damage was not observed in the mice following treatment with iMDK (9 mg/kg). Serum levels of AST and ALT were measured 48 hours after iMDK treatment as described in Methods. Shown is the mean of the AST and ALT from four mice of each group; bars, SD.
(TIF)

Figure S5 **PD0325901 did not alter endogenous MDK expression in H441 lung adenocarcinoma cells.** The MEK inhibitor (PD0325901) inhibited phosphorylation of ERK but not MDK in H441 cells. Shown is immunoblot performed as described in Methods.
(TIF)
