# Correction: Inhibition of the Growth Factor MDK/Midkine by a Novel Small Molecule Compound to Treat Non-Small Cell Lung Cancer

**DOI:** 10.1371/journal.pone.0307052

**Published:** 2024-07-09

**Authors:** Huifang Hao, Yutaka Maeda, Takuya Fukazawa, Tomoki Yamatsuji, Munenori Takaoka, Xiao-Hong Bao, Junji Matsuoka, Tatsuo Okui, Tsuyoshi Shimo, Nagio Takigawa, Yasuko Tomono, Motowo Nakajima, Iris M. Fink-Baldauf, Sandra Nelson, William Seibel, Ruben Papoian, Jeffrey A. Whitsett, Yoshio Naomoto

In Fig 5, the beta-actin is incorrect in panel B. Please see the correct Fig 5 here.

**Fig 5:**
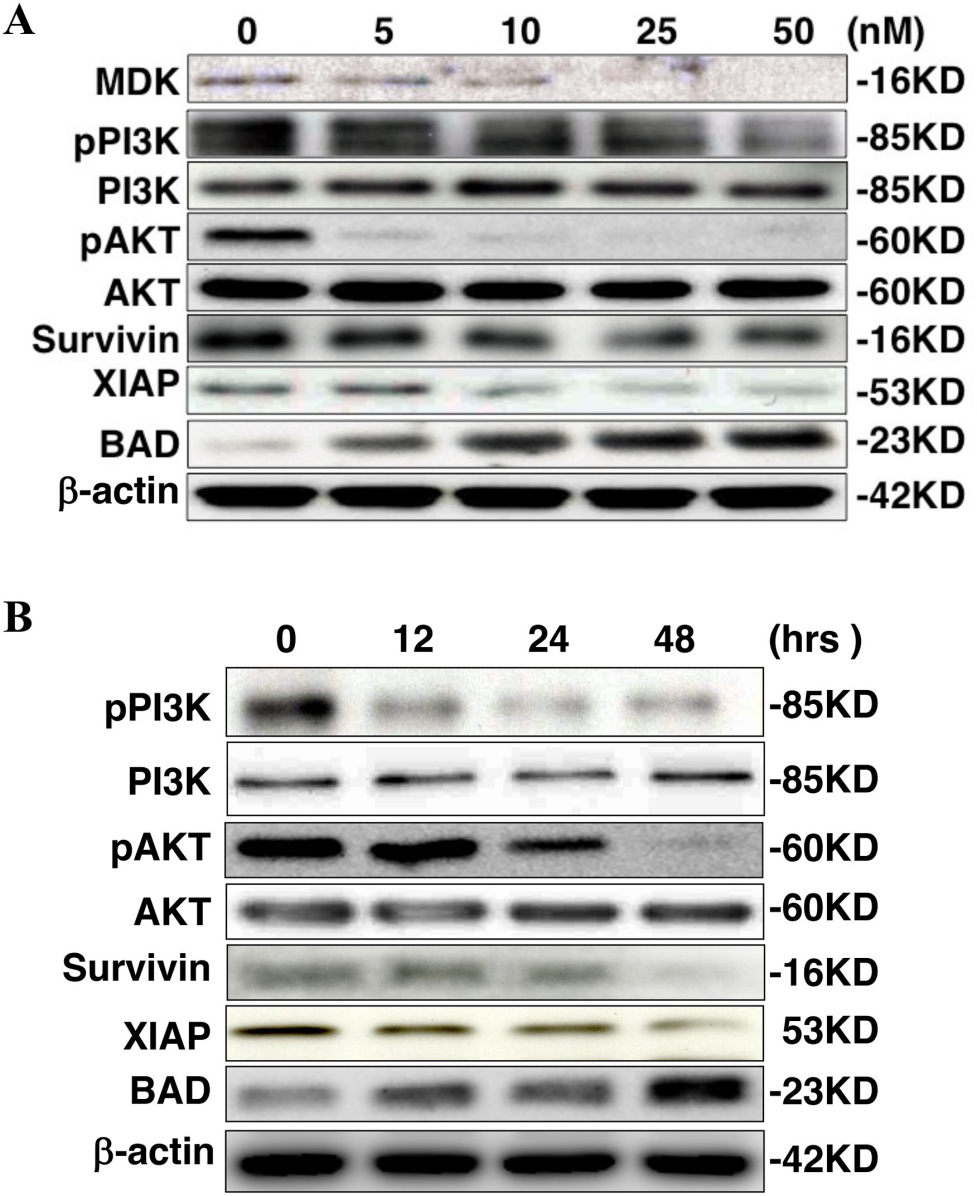
iMDK inhibited the PI3K/AKT pathway and influenced the apoptosis pathway. **A.** Dose-dependently, phosphorylation of PI3K and AKT and the expression of survivin and XIAP, anti-apoptotic factors, were decreased while the expression of BAD, a pro-apoptotic factor, was increased 48 hours after treatment with iMDK. Shown is immunoblot performed as described in Methods. **B.** Time-dependently, phosphorylation of PI3K and AKT and the expression of survivin and XIAP were decreased while the expression of BAD was increased by treatment with iMDK at a concentration of 50 nM. Immunoblot was performed as described in Methods.
