# Supplementary figures and images for: Inhibition of the Growth Factor MDK/Midkine by a Novel Small Molecule Compound to Treat Non-Small Cell Lung Cancer
Source: PLoS One. 2013 Aug 16;8(8):e71093. doi: 10.1371/journal.pone.0071093 (PMC3745462; doi:10.1371/journal.pone.0071093)

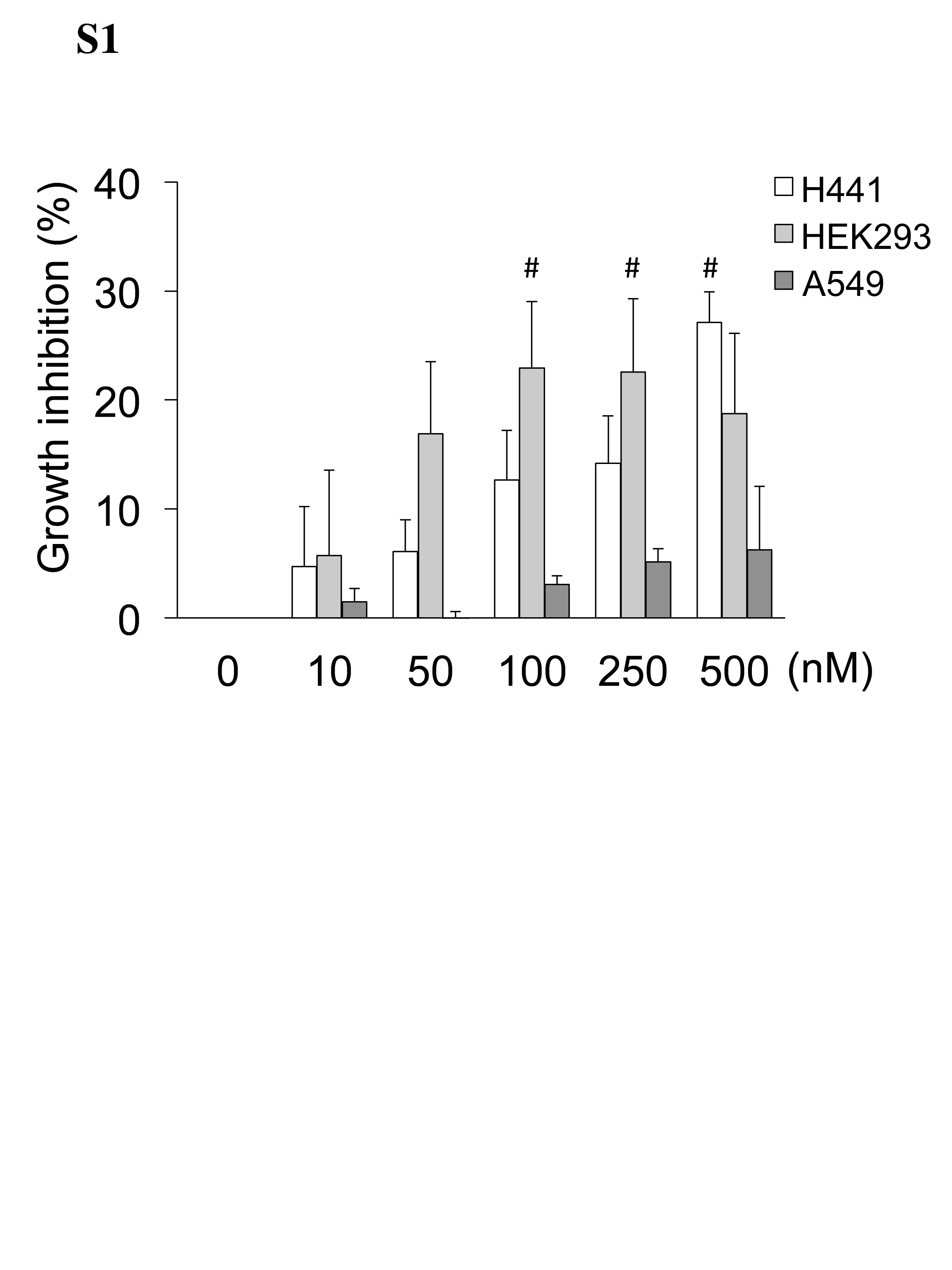

Supplement: Figure S1 — Growth inhibition by iMDK was increased in MDK-expressing cells. iMDK induced growth inhibition in MDK-positive H441 lung carcinoma cells and HEK293 embryonic kidney cells but not in MDK-negative A549 lung carcinoma cells. Cell viability was assessed by the WST-1 assay 48 hours after the iMDK treatment and represented as % growth inhibition as described in Methods. Statistical significance was defined as p<0.05 (#). (TIF) [file pone.0071093.s001.tif]

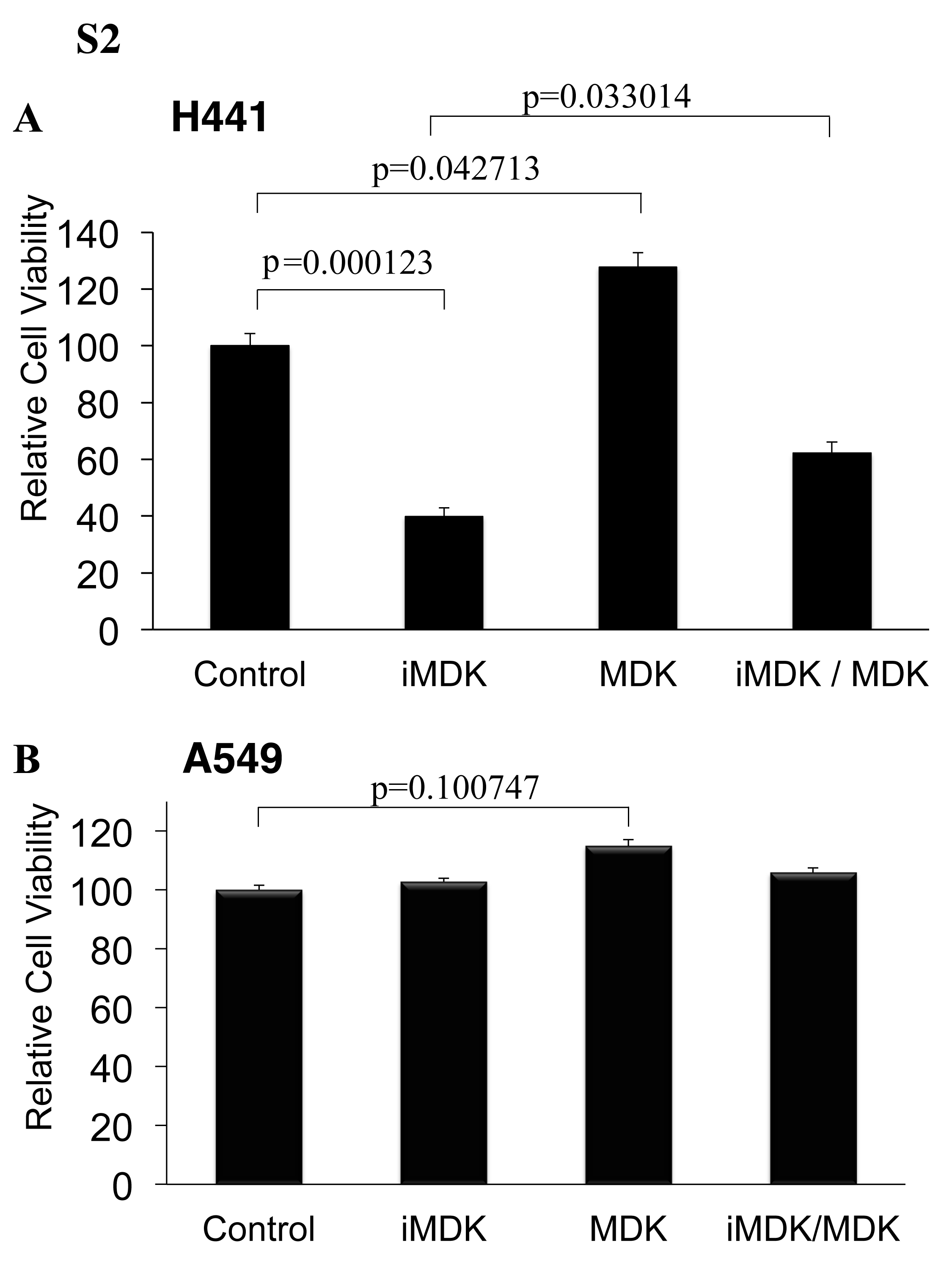

Supplement: Figure S2 — Recombinant MDK rescued iMDK-induced cell viability inhibition in H441 lung adenocarcinoma cells. A. Recombinant MDK (25 nM) blocked the iMDK (25 nM)-mediated cell growth inhibition in MDK-positive H441 lung adenocarcinoma cells. Cell viability was assessed by trypan blue exclusion assay as described in Methods. B. Recombinant MDK (25 nM) and/or iMDK (25 nM) did not alter cell growth in MDK-negative A549 lung carcinoma cells. Cell viability was assessed by trypan blue exclusion assay as described in Methods. (TIF) [file pone.0071093.s002.tif]

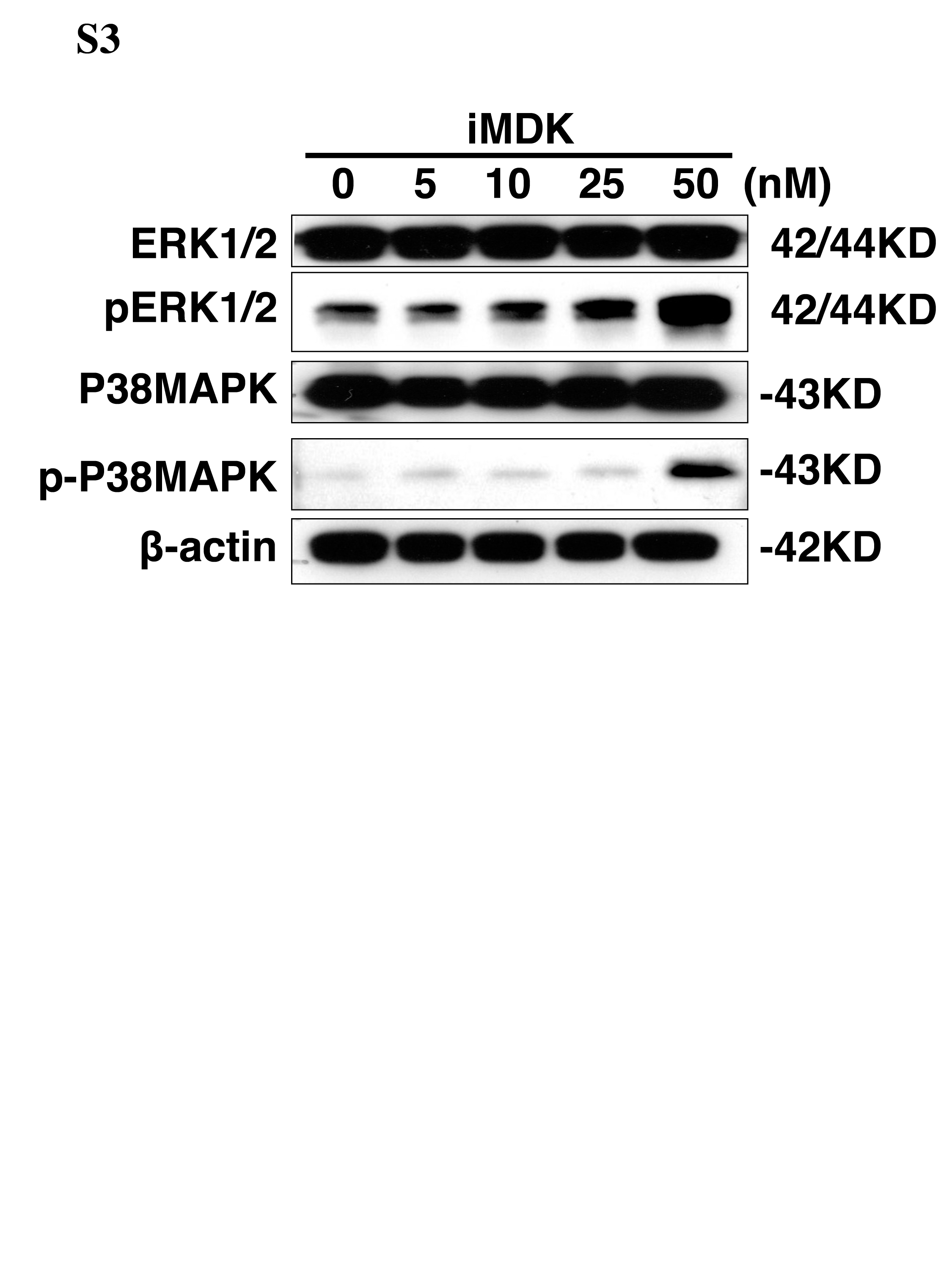

Supplement: Figure S3 — iMDK activated the MAPK pathway in H441 pulmonary adenocarcinoma cells. Phosphorylation of ERK (a MAPK) and p38MAPK was increased 48 hours after treatment with iMDK at the indicated concentrations in H441 cells. Shown is immunoblot performed as described in Methods. (TIF) [file pone.0071093.s003.tif]

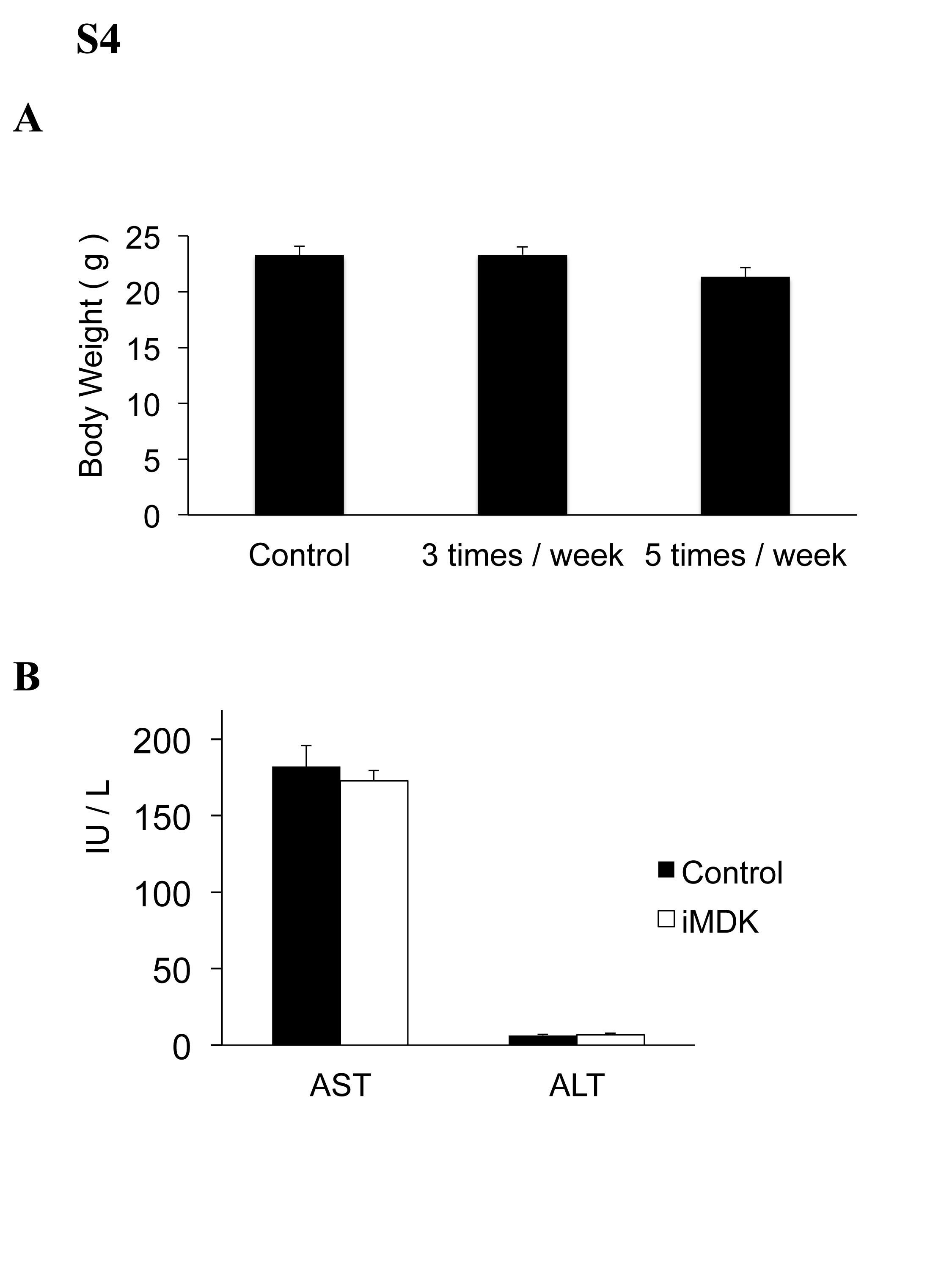

Supplement: Figure S4 — Systemic toxicity was not observed after iMDK treatment in BALB/c nude mice. A. Body weight of the nude mice was not altered by treatment with iMDK (9 mg/kg). The body weight of each mouse group (DMSO administered Control, 3 times/week or 5 times/week) was measured on day 10. Shown is the mean of the body weight (g) from four mice of each group; bars, SD. B. Liver damage was not observed in the mice following treatment with iMDK (9 mg/kg). Serum levels of AST and ALT were measured 48 hours after iMDK treatment as described in Methods. Shown is the mean of the AST and ALT from four mice of each group; bars, SD. (TIF) [file pone.0071093.s004.tif]

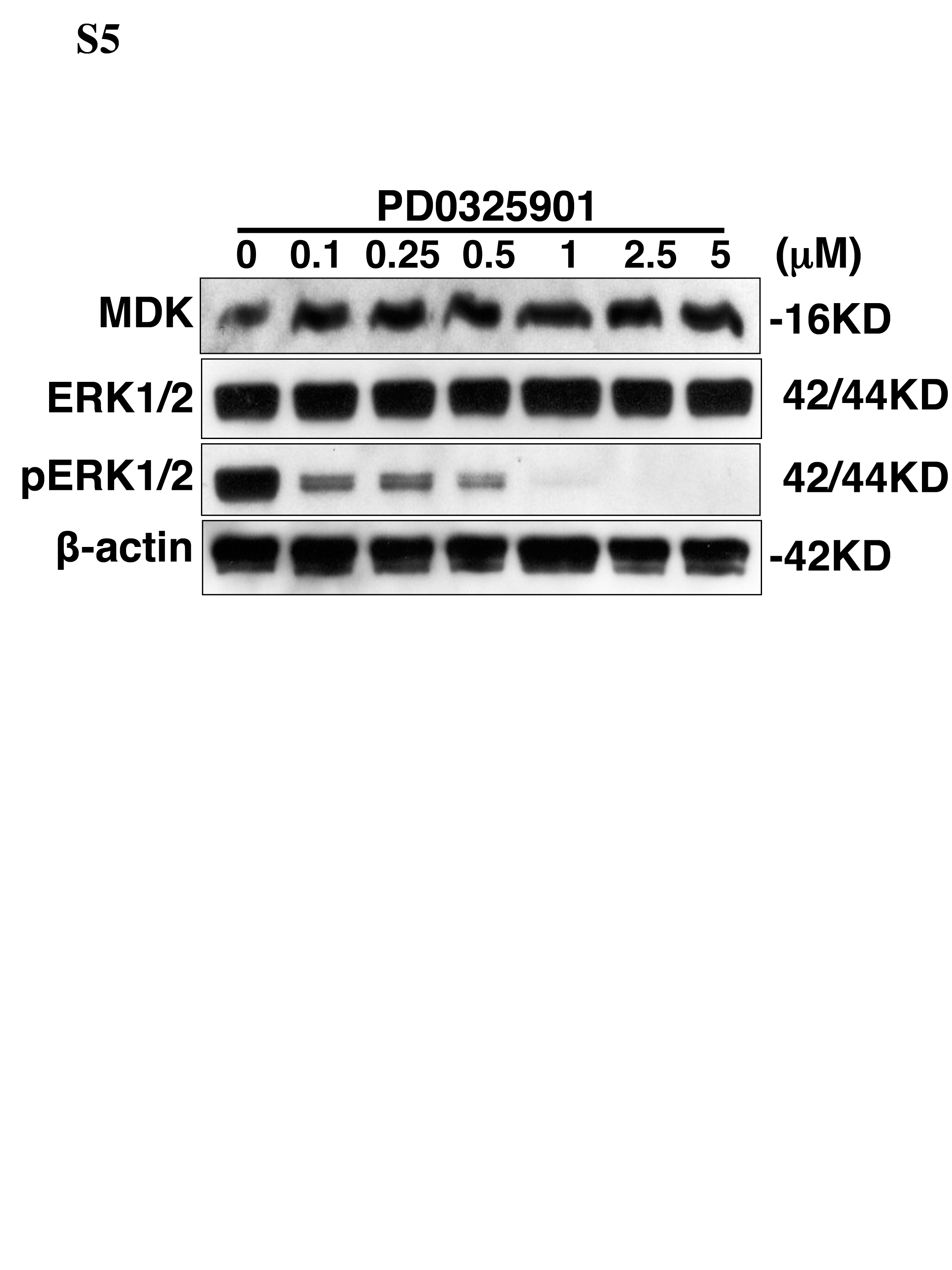

Supplement: Figure S5 — PD0325901 did not alter endogenous MDK expression in H441 lung adenocarcinoma cells. The MEK inhibitor (PD0325901) inhibited phosphorylation of ERK but not MDK in H441 cells. Shown is immunoblot performed as described in Methods. (TIF) [file pone.0071093.s005.tif]
